# Control and Real-Time Data Acquisition of an Experimental Platform for Stored Grain Aeration Study

**DOI:** 10.3390/s21165403

**Published:** 2021-08-10

**Authors:** Jingyun Liu, Ping Li

**Affiliations:** College of Urban Rail Transit and Logistics, Beijing Union University, Beijing 100101, China; zdhtliping@buu.edu.cn

**Keywords:** control, data acquisition, stored grain aeration, experimental platform

## Abstract

Aeration is one of the most important methods to keep stored grain safe and maintain its quality. Experimental platforms are used for stored grain aeration study in a laboratory-scale. The purpose of this paper was to provide the real-time data acquisition and control system design of a new experimental platform with multifunction for stored grain study. Requirements of the aeration experiments were analyzed, and multi running modes were designed. The aeration inlet air conditions were designed to be adjustable and multi variables need to be controlled simultaneously, which was a key problem to be solved for the platform. An ON/OFF-PID based multivariable cooperative control method was proposed, and two control loops were formed where inlet air temperature and humidity were considered separately while could be controlled simultaneously with a logic judgement strategy. Real-time data needed to be monitored was acquired with different sensors and displayed intuitively. Experiments were carried out to test the static and dynamic characteristics of the control method and three inlet air flow rates of 0.03, 0.08 and 0.13 m·s^−1^were used. Performance of the data acquisition system was also tested. The results showed that, the inlet air conditions control error was within ±1 °C and 10% for temperature and relative humidity, respectively. The real-time data acquisition of multi parameters during aeration process was realized. The experimental platform can be used for studies of different aeration objectives.

## 1. Introduction

Grain storage is very essential for food supply, especially in 2020 when the COVID-19, plague of locusts, and the frequent occurrence of natural disasters threatened stable growth of grain production. Advanced grain storage technologies have become particularly important. The temperature and moisture content are the most important factors for grain storage [1,2]. The population of insects and mites, fungal growth and grain quality are in close relation with these two factors [3,4,5,6]. Aeration is the key grain storage technology [7,8], which can be used to maintain grain safety and quality by adjusting the grain temperature and moisture content [9]. Heat, mass and momentum transfer occurs during the aeration process [10], which is mainly determined by the original grain temperature and moisture content, the inlet air temperature, humidity and the flow rate, and the outside environment if the bin is not insulated. Conditions of these parameters will influence not only the final grain temperature and moisture distribution, but also the aeration process, in terms of heat, mass and momentum transfer speed, energy consumption, and even the grain quality. It is very important to study the aeration mechanism and control for grain storage management [11]. Therefore, mathematical models are developed by researchers to better understand and simulate the stored grain aeration process [12], and control strategies are proposed for stored grain aeration management [13].

Many of these studies are presented in literatures, and model simulations and experiments have been carried out [14,15,16,17,18]. Experiments can be used to reveal important phenomenon and validate newly developed models and control methods, which provides important means for stored grain aeration study. Experiments run in field grain storage bins are rational and more likely to achieve the reliable results. However, they are usually time and labor consuming, and much more grain is needed, which may result in massive waste if the experimental process is not well managed. Sometimes, the required conditions are rather difficult to reach in field experiments. The experiment in a laboratory scale is easy to be carried out and more focuses on the specific problem by eliminating irrelevant factors. That is why many studies of stored grain are executed on an experimental platform [8,12,18,19,20,21,22,23]. 

Most of the aeration platforms under laboratory conditions can be classified into two types.

One type involves using the integrated air supply system and the simulated storage bin. The inlet air can be the ambient air without pretreated process and blown into the bin directly or with specially designed control strategies [8]. The inlet air can also be heated for in-bin drying [20] or cooled for cooling the stored grain [21,22]. The inlet air is rarely pretreated for different aeration objectives. The bins are usually instrumented with sensors [8,20,21,22]. This type of experimental platform usually has simple inlet air treated systems which makes it easy to construct. It is usually designed for one or two aeration objectives, unable to meet various aeration study requirements. 

The other type of platform is using artificial climate chamber to simulate different aeration inlet air conditions, which can be used for different aeration objectives [12,18,23]. The bin instrumented with sensors for data acquisition is placed inside the chamber, and inlet air condition is the same as the chamber inner environment. The cubic bin and column bin are used. In [12], the authors designed a new aeration system with three bins where the three-replicate data were collected in one aeration process. For this kind of platforms, control method is required for the chamber to obtain the desired aeration conditions. Sometimes the inlet air conditions might be influenced by the fluctuations of the chamber environment. That was probably because the air temperature, humidity and velocity fields distribute non-uniformly inside the chamber, which is difficult to be eliminated. For this type of platforms, the chamber construction might be the most complex and costly part. It will also take up considerable space. 

In addition to the existing functions on the above aeration experimental platforms, the real bin aeration requirements should also be considered to develop more suitable platforms for aeration study in a laboratory scale. Some aeration tests are executed in real bins. During cooling-aeration process, it is required to minimize the grain moisture content loss [24]. Therefore, the constrained inlet air condition control study experiments (either using the adsorption and desorption model [24] or equilibrium moisture content model [18]) should be considered in the platform design. For the ambient air aerations, turning on and off of the fan can be performed manually or automatically. If inlet air is pretreated, automatic control of the heating, refrigeration or humidifying will need to be realized. Real-time data acquisition is also essential in real bins [25]. 

A multi-function experimental platform in the laboratory scale is more likely to be an effective solution for various aeration studies. The following aspects should be considered:

(1) Aeration experiments using ambient air or pretreated air with constant or varied flow rate can all be conducted on the platform.
(2) For pretreated air aeration, the control method can realize stable and reliable inlet air conditioning.
(3) Real-time data of multi-sensor parameters should be acquired.
(4) The platform can be more compact, have low construction cost, supply air for different bins, and be easy to reproduce.

A new multi-functional experimental platform for different stored grain aeration needs was designed in this work. It could be used for both the mechanism study, which aimed at better understanding the parameter changes during aeration process; and the control study, which emphasized the process optimization. The automatic control and the real-time data acquisition were realized, which would be introduced in detail. 

This paper was organized as follows: First, requirements of stored grain aeration were analyzed. Then the platform design details were introduced. Finally, tests were carried out to validate the performance of the platform and the platform control characteristics were analyzed.

## 2. The Laboratory-Scale Experimental Platform for Stored Grain Aeration Study

### 2.1. Monitoring and Control Requirements of the Stored Grain Aeration Experimetal Platform 

**Figure 1:**
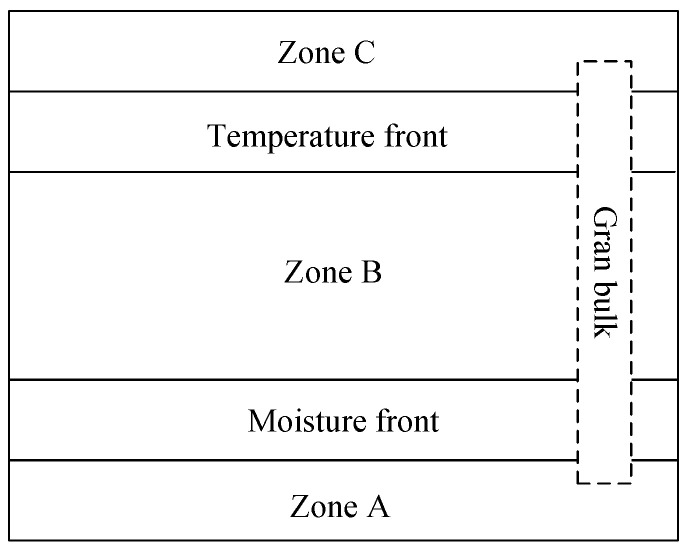
Three zones and two fronts of the grain bulk during aeration with constant and uniform inlet air conditions.

**Figure 2:**
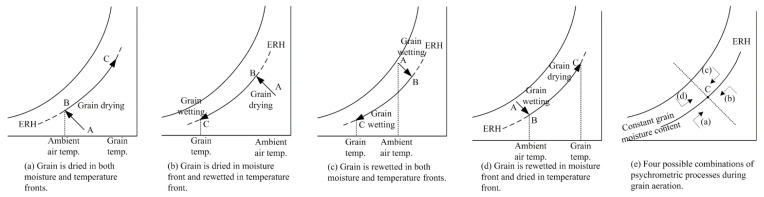
Possible psychrometric processes during aeration.

During the aeration of stored grain, air was forced through the grain bulk with a mechanical fan. For different objectives, the inlet air may be heated, cooled, humidified, dehumidified or not treated. The inlet air temperature, humidity and flow rate would influence the drying, cooling, rewetting rate and final grain hygrothermal parameters. The heat and mass transfer could possibly be explained like the adiabatic drying process. If blown through with a uniform air rate, and constant temperature and relative humidity, the grain bulk would form three zones (A, B, C) and two fronts (temperature front and moisture front) as shown in Figure 1 [26]. In zone A, the intergranular air was at the inlet air conditions and the grain was in equilibrium with the inlet air. In the moisture front, it was an adiabatic process during which air provided the heat to evaporate the moisture of the grain. In zone B, the grain temperature was near or between the dry bulb and wet bulb temperature of the air. In the temperature front, the air got into equilibrium with the initial grain conditions. In zone C, the grain was at the initial temperature and moisture content. Possible psychrometric processes could be shown in Figure 2. ERH was the equilibrium relative humidity. Monitoring the conditions of the grain and intergranular conditions during aeration was rather essential to learn about the proceedings. If the inlet air conditions changed, which usually happened in the real field, the grain and intergranular air conditions inside the bin would become more complicated, and a good knowledge of this process would be helpful to work out appropriate management measures. For the experimental platform design, various aeration objectives should be considered. Inlet air could be used for aeration management study with low cost. In some cases, the ambient air was heated before flown into the bin such as in-bin drying. Moreover, it was important to supply constant inlet air conditions with good accuracy for certain mechanism study, and also varied inlet air conditions for different aeration control method studies during an aeration process. Speed of this process could be affected by the air flow rate. Therefore, the inlet air temperature, humidity and flow rate of laboratory-scale experimental platform was designed to be adjustable for different aeration objectives or left untreated. The room air temperature and the inlet air pressure were also needed to be recorded. Therefore, the stored grain conditions, the inlet air conditions and environment air conditions were monitored, and real-time data were recorded and stored online. Grain moisture content would be measured off-line.

### 2.2. Structure of the Platform

**Figure 3:**
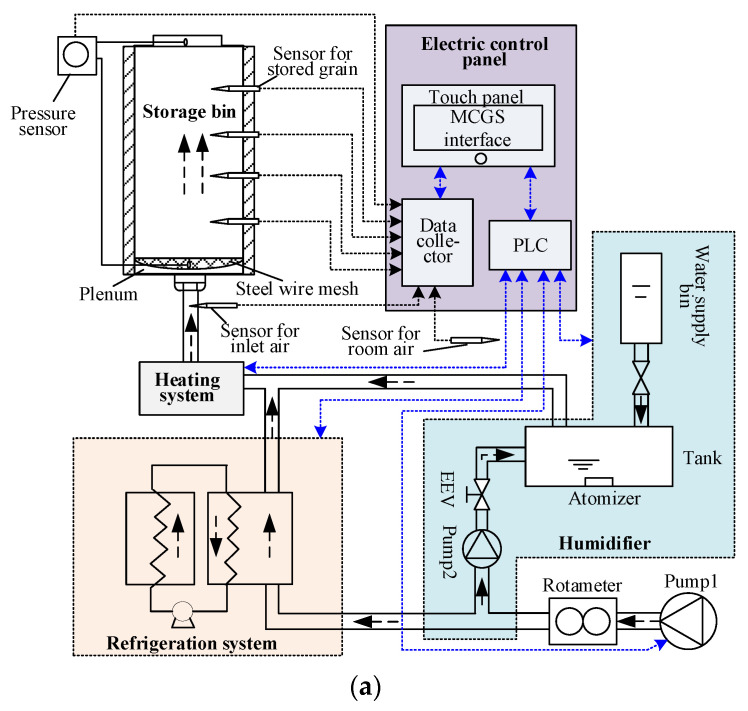

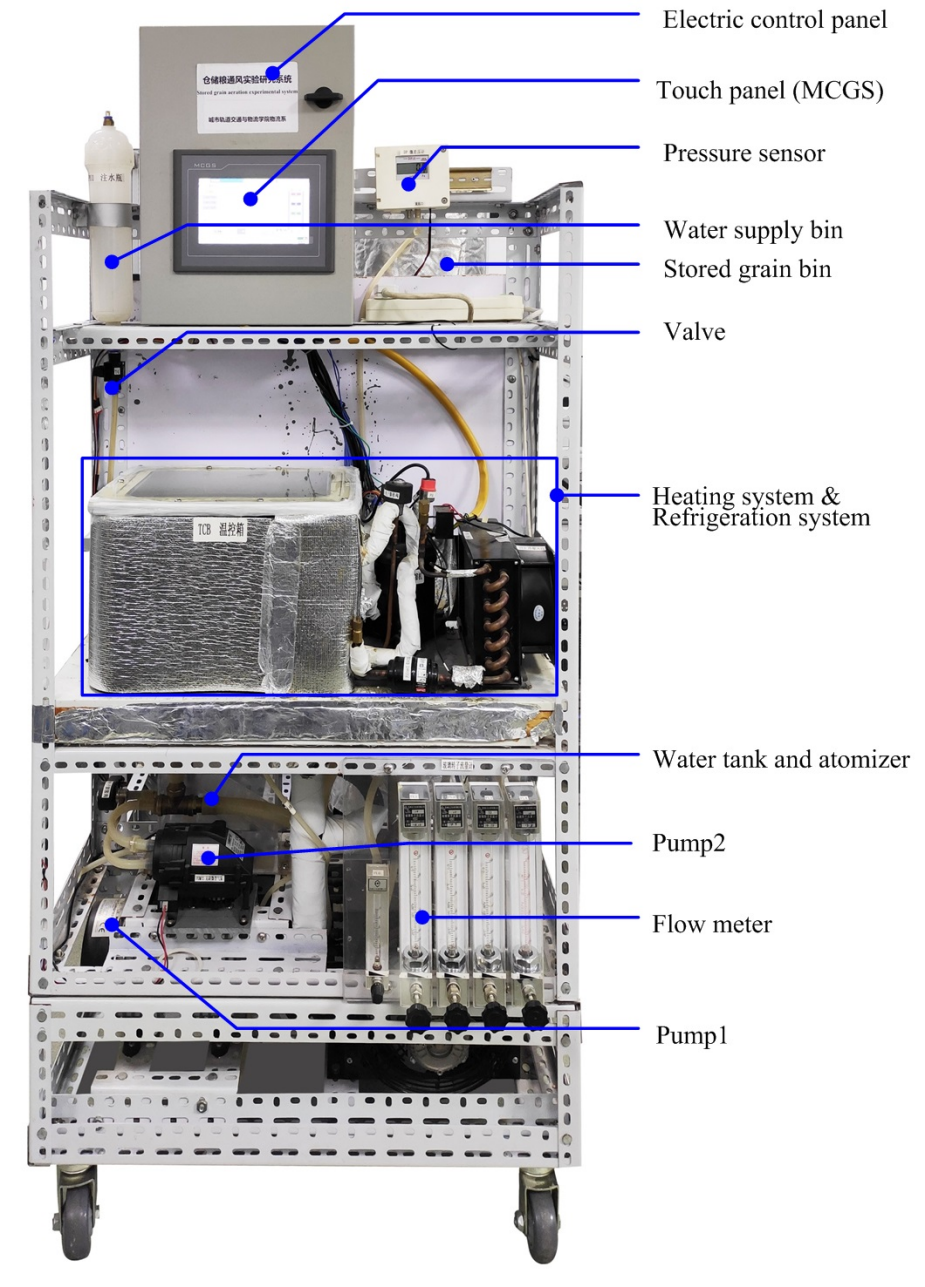
Structure and picture of the experimental platform: (**a**) Structure of the platform; (**b**) picture of the platform.

To meet the requirements of the stored grain aeration process study, the experimental platform was designed with a structure shown in Figure 3a, and its picture was shown in Figure 3b. It was mainly composed by the pump, rotameter, humidifier, refrigeration system, heating system, sensors, electric control panel and the storage bin.

As illustrated in Figure 3, the ambient air was blown into the air supply system by pump1 (AC0-380, Guangdong Hailea Group Co., Ltd., Chaozhou, China) and its volume could be adjusted with four LZB-10 glass rotameters (2500 L/h, Suzhou Chemical Instrument Co., Ltd., Suzhou, China) and one LZB-6WB glass rotameter (600 L/h, Suzhou Chemical Instrument Co., Ltd., Suzhou, China). Then, the air was cooled and dried by the refrigeration system which consisted of a compressor (BSA357CV-R1AN, Hitachi Manufacturing Co., Ltd., Tokyo, Japan), an evaporator (FE0, Beijing Creative Communication Technology Co., Ltd., Beijing, China) and a condenser (FC0, Beijing Creative Communication Technology Co., Ltd., Beijing, China). The humidifier was composed by pump 2 (FCY5015-24V, Chengdu Xinweicheng Technology Co., Ltd., Chengdu, China), electronic expansion valve (EEV) (TS130CV, Saginomiya Seisakusho, INC., Tokyo, Japan), atomizer (SH0, Beijing Creative Communication Technology Co., Ltd., Beijing, China), a tank and the water supply bin. If the inlet air needed to be humidified, the EEV would be opened and the inlet air was bypassed by pump2, and the air volume was controlled with EEV. The air from the humidifier and the refrigeration system joined and then flew into the heating system (Electronic heaters H0, Beijing Creative Communication Technology Co., Ltd., Beijing, China). At the end of the air supply system, temperature and relative humidity of the air flown into the bin was measured with SHT11 (Sensirion AG, Staefa, Switzerland) and used as feedbacks for control. Four holes were punched for sampling stored grain to measure the moisture content. The intergranular air temperature and relative humidity could be measured with four SHT11 sensors at the same heights of the holes. The pressure difference between the inlet and outlet air was measured with a pressure sensor (DP102, Beijing North Dahe Instrument Co., Ltd., Beijing, China). A touch panel (TPC7062KX, Shenzhen Kunluntongtai Technology Co., Ltd., Shenzhen, China) was used to embed the control method and display the real-time data gathered by a data collector (BEC-2103, Beijing Creative Communication Technology Co., Ltd., Beijing, China) from the sensors, and sent the control commands to a PLC (FP0R-C14RS, Panasonic Corporation, Osaka, Japan), which would control the instruments and elements in turn as shown in Figure 3.

### 2.3. Running Mode Design of the Platform

To meet different stored grain aeration requirements, three running modes were designed for the platform. They were the manual mode, T mode and T&H mode. In the manual mode, the ambient air was used for aeration, during which only inlet air flow rate could be adjusted. This mode could be used for ambient air aeration control studies. In the T mode, inlet air temperature could be controlled to the desired value with varied flow rate. This mode could be used for in-bin drying or cooling. In the T & H mode, inlet air temperature, relative humidity could be controlled with varied air flow rate, which could be used for various aeration objectives, such as model validation, advanced control methods and so on. The modes could be chosen through the platform interface which would be introduced in detail in Section 2.6.

### 2.4. Parameters Range Design for the Inlet Air 

The inlet air temperature and relative humidity could be controlled for different aeration objectives, such as cooling, drying or rewetting the grain. All these aeration objectives were for the purpose of changing the grain temperature and moisture content to a certain level for safe or economic grain storage. Inlet air temperature and humidity would determine the final grain temperature and moisture content. Inlet air of low temperature was used for cooling, and the air relative humidity was also calculated with the desired equilibrium moisture content of grain to avoid over drying or over wetting. During the drying process, the heated air was usually used, and the grain moisture content would be reduced gradually which took a rather long time. After drying, the grain temperature was usually high, if not cooled down in time, grain rewetting might happen. Aeration could also be used for rewetting the stored grain for economic reasons and this process needed to be controlled more strictly for grain safety. The aeration process consumed a lot of energy, which could be optimized by setting appropriate inlet air parameters with no harmful influence on the grain security and quality [18]. That is the reason why the inlet air temperature and relative humidity were designed to be changeable on the experimental platform for different aeration objectives. The inlet air temperature range was from 10 to 40 °C, and the relative humidity from 20% to 80%.

The inlet air flow rate also influences the aeration process. Bartosik [27,28] studied the influence of three inlet air velocities and used the optimized option in the in-bin drying study. Under a higher inlet air flow rate, more heat and mass would be transferred between grain and air per unit time except when the drying or the cooling process was governed by the mass or heat transfer speed inside grain kernels. However, a higher flow rate usually meant more energy consumption. If the objectives, such as the grain security, quality and energy saving were taken into account, there would be an optimized flow rate for certain aeration objective. Therefore, the inlet air flow rate in the experimental platform was also changeable. The air volume flow rate was 0~176 L/min, which can be set with the flow meter. Different size of bins could be used for aeration. For example, if the diameter of bin was 0.15 m, then the inlet air flow rate range was from 0 to 0.166 m/s.

### 2.5. Control Method of the Inlet Air Temperature and Relative Humidity 

In the T mode, the inlet air temperature could be controlled. In the T&H mode, both the inlet air temperature and relative humidity were controlled automatically. For the T mode control was part of the T&H mode control, the latter would be introduced in detail.

**Figure 4:**
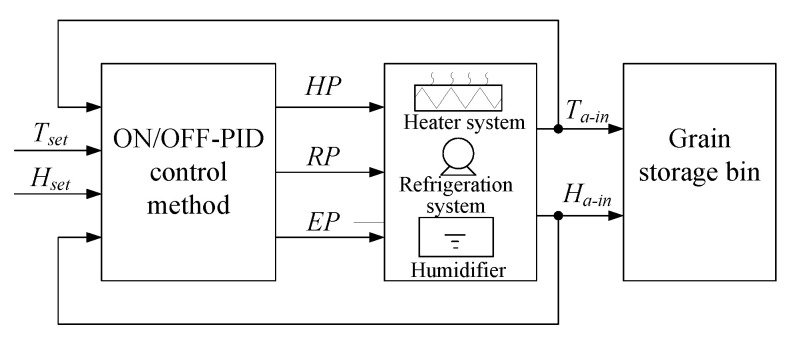
The platform control architecture.

For the T&H mode control, the platform had multi-controlled variables, such as the inlet air temperature *T_a-in_* and relative humidity *H_a-in_*, which were coupled with each other. An ON/OFF-PID (ON/OFF and proportional integral derivative) based multi-variable cooperative control method was proposed. The control architecture was shown in Figure 4. Set points of *T_a-in_* and *H_a-in_* were *T_set_* and *H_set_*, respectively. The control variables were heating percent *HP* and refrigeration percent *RP* and EEV opening rate *EP* (used to adjust the humidification capacity). 

The ON/OFF control and PID control were used in the control method. The control law of PID was shown in Equation (1) and its discrete form was used to develop the controller [29],

(1) $$u(t)=k_{p}(e(t)+\frac{1}{T_{i}}\int_{0}^{t}e(t)dt+\frac{T_{d}de(t)}{dt})$$

where *e*(*t*) was the difference between the set point and the real-time value of the controlled variables in time step *t*; *K_p_* was the proportional coefficient, *T_i_* was the integral time constant, *T_d_* was the differential time constant. *K_p_*, *T_i_* and *T_d_* were obtained with engineering design method. *K_p_* was determined first. *K_p_* was increased until the system oscillated while *T_i_* and *T_d_* was set to zero. Then *K_p_* was decreased until the oscillation disappeared and set *K_p_* as 60–70% of the current value. Then, *T_i_* was set to a large initial value and decreased until oscillation appeared. The next step was to decrease *T_i_* until the oscillation disappeared and set *T_i_* to 150~180% of the current value. *T_d_* was obtained with the same method. Finally, the three parameters were fine-tuned until the system was well controlled.

**Figure 5:**
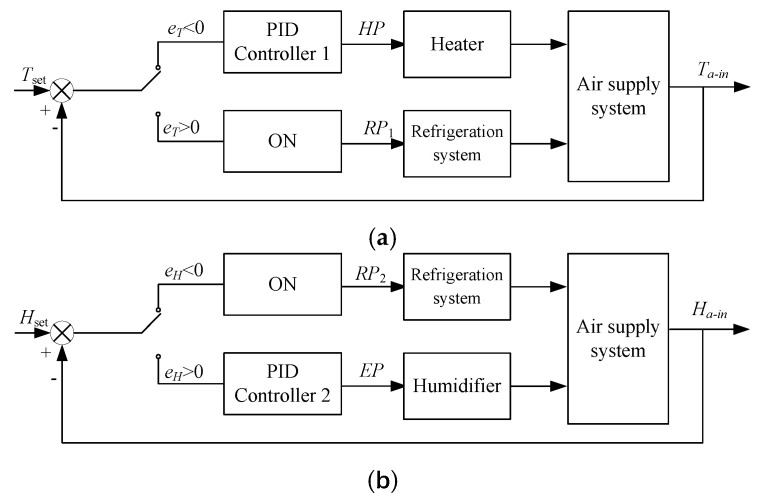
Control scheme of the experimental platform: (**a**) The inlet air temperature control scheme; (**b**) The inlet air relative humidity control scheme.

The control scheme was shown in Figure 5. Two control loops were designed for inlet temperature and relative humidity respectively. In the T mode, only the T control loop was used. In the T&H mode, the control variables for *T_a-in_* were *HP* and *RP*_1_, and for *H_a-in_* were *EP* and *RP*_2_*. HP* was calculated with PID controller 1, and *EP* was calculated with PID controller 2. *RP* was obtained with the ON/OFF control method, and the ON condition was determined by both the inlet air temperature error (*e_T_*, Equation (2)) and relative humidity control error (*e_H_*, Equation (3)):

(2) $$e_{T}=T_{set}-T_{a-in}$$

(3) $$e_{H}=H_{set}-H_{a-in}$$

If *T_a-in_* was lower than *T_set_* (*e_T_* > 0), the heater would work and *HP* was calculated. The refrigeration system would not work and *RP*_1_ 0. If *e_T_* < 0, the heater would not work and *RP*_1_ 100%. If *H_a-in_* was higher than the set point (*e_H_* < 0), the refrigeration system was used to dehumidify the air and the *RP*_2_ 100%. If *e_H_* > 0, the humidifier would work and *EP* would be calculated and *RP*_2_ 0. If *RP*_1_ > *RP*_2_, *RP*_1_ would be used to adjust the refrigeration system (*RP RP*_1_), else the *RP*_2_ would be used (*RP* = *RP*_1_).

**Figure 6:**
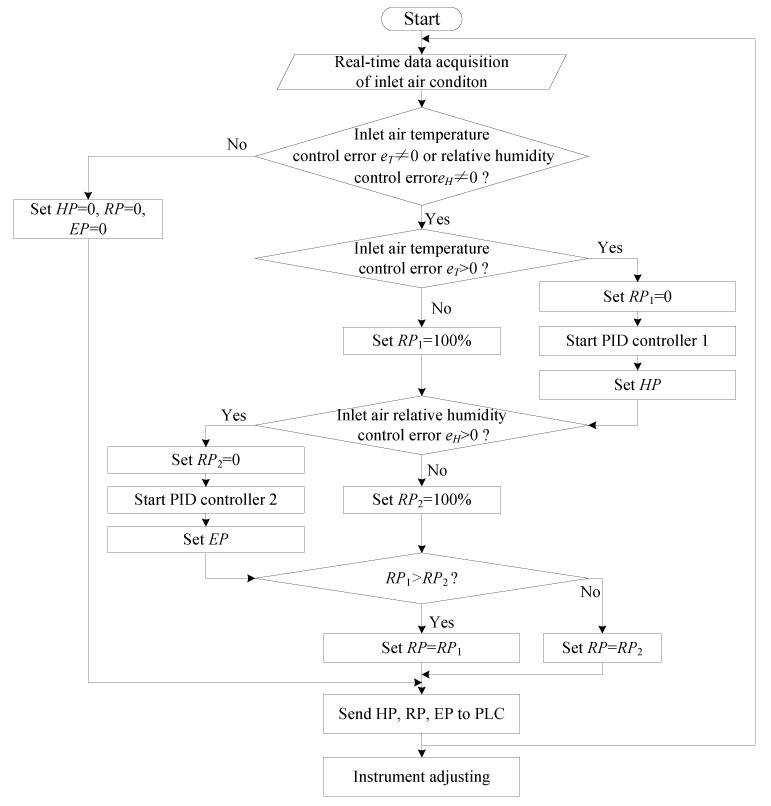
Control flow chart.

The control logic of the proposed control method was presented in a flow chart as shown in Figure 6.

### 2.6. Monitoring Interface of the Platform

**Figure 7:**
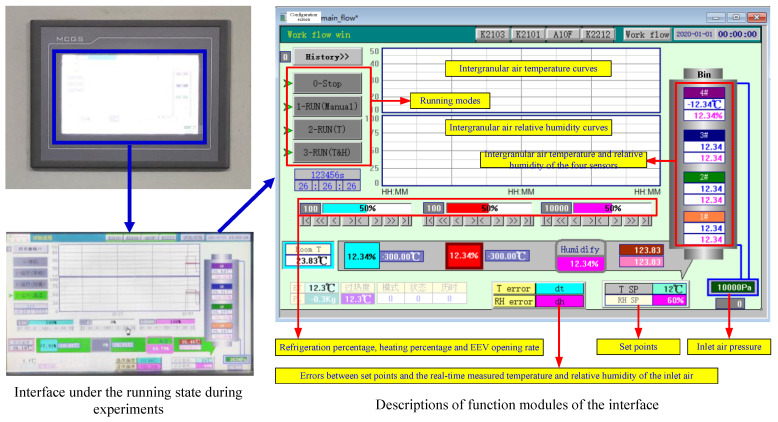
Touch panel interface of the experimental platform.

A monitoring interface embedded in the touch panel (shown in Figure 7) was developed with MCGS 7.7, a configuration software (Embed version, Shenzhen Kunluntongtai Technology Co., Ltd., Shenzhen, China), functions of which were annotated in detail in Figure 6. The three running modes could be chosen on the upper-left corner of the interface. Since the panel was a digital embedded system with a high-speed calculation capability, the control method introduced in Section 2.5 was also realized in MCGS through programming. The conditions of the inlet air and intergranular air inside the bin could be collected and monitored. *T_a-in_* and *H_a-in_* could be set on the interface and the errors between the measured parameters and the set points would be calculated, displayed and used as the input of the controllers. Therefore, the data and parameter states could be displayed on the panel and the system control task was executed as a background job of the panel. Moreover, the outputs of the controller were sent to the PLC which would drive the actuators of the system. 

## 3. Tests of the Experimental Platform

In order to validate the performance of the experimental platform, several tests were undertaken. 

### 3.1. Inlet Air Temperature and Relative Humidity Control Tests

The inlet air temperature, relative humidity and the air flow rate for stored grain aeration would determine the aeration process and the final grain temperature and moisture content. Tests for the inlet air temperature and relative humidity control (T&H mode) were carried out. For wheat, the stored grain moisture content was set at about 13% d.b., in accordance with which the inlet air temperature and relative humidity set points were determined by Equation (4) (the Chung-Pfost equation [30]) without considering the grain hysteresis state. This equation described the relationship between the grain equilibrium moisture content *M_ge_* and the air conditions,

(4) $$M_{ge}=-\frac{1}{C_{3}}\ln[\frac{(T_{a}+C_{2})\ln H}{-C_{1}}]$$

where, *T_a_* was the air temperature in °C; *H* was the air relative humidity in %. *C*_1_, *C*_2_, *C*_3_ were constants obtained from experiments. For wheat, *C*_1_ = 660, *C*_2_ = 220, *C*_3_ = 13.8.

*T_a-in_* and *H_a-in_* were measured with SHT11 sensor. The temperature measuring range was −40~123.8 °C, with accuracy of ±0.4 °C at 25 °C, and the relative humidity measuring range was 0~100% RH, with accuracy of ±3%.

Three inlet air flow rate of 0.03 m·s^−1^, 0.08 m·s^−1^ and 0.13 m·s^−1^ were used for the tests. The average pressure differences between the inlet and the outlet air for the three flow rates were 778, 1997 and 3100 Pa respectively, measured with the pressure sensor (0~10 KPa in range, with accuracy of ±1% full scale at 20 °C). The ambient air temperature was 22 °C and relative humidity was 45%. The set points of the inlet air parameters for the tests were set at 35 °C/61% and 15 °C/60%, which were constrained by Equation (4).

### 3.2. Real-Time Data Acquisition Tests

Real-time data was essential for future aeration modeling and control study. The stored grain cooling test was conducted on the platform to test the data acquisition function. The stored grain intergranular air temperature and relative humidity were measured with the SHT11. The grain storage bin used in the test was a PVC pipe (0.15 m in diameter, 1 m in height, 0.001 m in thickness of the bin wall). Four holes were punched along the vertical direction of the bin, through which connectors (0.016 m in inner diameter) were fixed for the sensors and sampling pipes which would be plugged in and pulled out easily. For better heat insulation, a layer of foaming NBR with a thickness of 0.055 m was used to cover the outside of the bin, and the thermal conductivity of the material is 0.035~0.038 W/m °C. At the bottom of the bin, a steel wire mesh was fixed to bear the grain and form a plenum together with the bottom cover (as shown in Figure 3). Two holes were penetrated in the bottom cover, where a connector with inner diameter of 0.016 m and a smaller connector of 0.005 m were fixed for the inlet air and pressure sensor respectively. A rubber pipe with insulated outside was used to connect the inlet air connector and the air supply system. All the connection parts of the bin were designed following the uniform size standard. So, it was very easy to change the bin to another size according to different experimental requirements, while using the same air supply system. All the real-time data acquired would be transferred to the touch panel (as shown in Figure 3) and displayed on the MCGS interface (as shown in bin part of Figure 6) every 30 s. The changing curves of the measured data could also be displayed.

Wheat with initial moisture content of 10% was filled in the bin and the height was 0.95 m. The average initial intergranular air temperature was about 22 °C and relative humidity was 54.7%. The inlet air temperature and relative humidity was set at 17 °C and 60% respectively, which would not change the wheat moisture content a lot in order to observe the changing trends of the temperature and humidity inside the bin. The inlet air flow rate was set at 0.13 m·s^−1^. Because the temperature and relative humidity of the inlet air flown into the bin was the directly controlled variables, inlet air conditions would be more stable and reliable during the tests.

## 4. Results and Discussion

### 4.1. Inlet Air Temperature and Relative Humidity Control Test Results and Discussion

**Figure 8:**
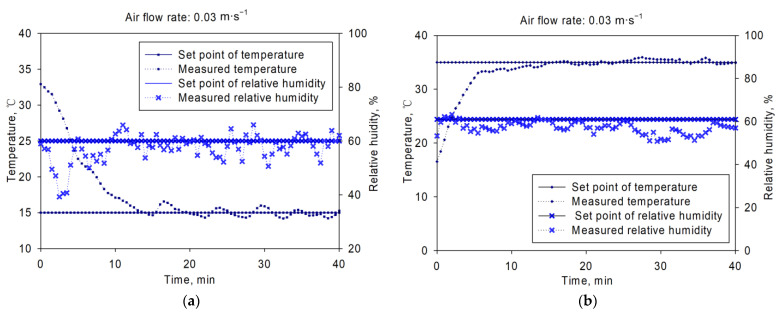
Temperature and relative humidity control results under air flow rate of 0.03 m·s^−1^: (**a**) with set point of 15 °C/60%, (**b**) with set point of 35 °C/61%.

**Figure 9:**
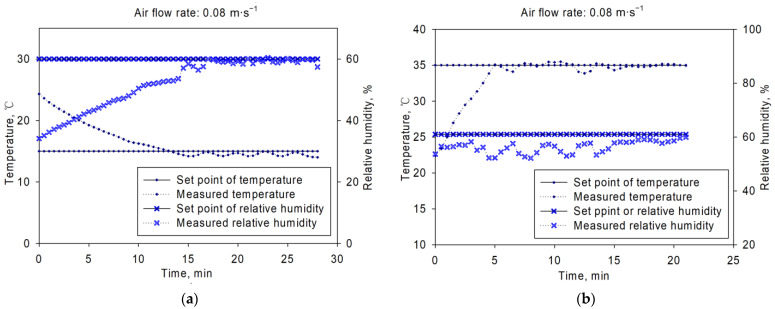
Temperature and relative humidity control results under air flow rate of 0.08 m·s^−1^: (**a**) with set point of 15 °C/60%, (**b**) with set point of 35 °C/61%.

**Figure 10:**
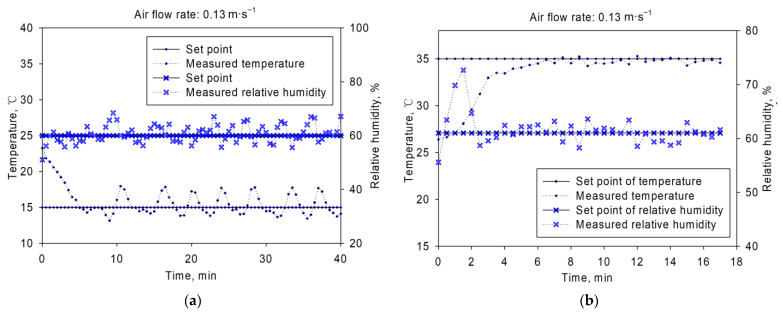
Temperature and relative humidity control results under air flow rate of 0.13 m·s^−1^: (**a**) with set points of 15 °C/60%, (**b**) with set points of 35 °C/61%.

The test results were shown in Figure 8, Figure 9 and Figure 10. Figure 8 showed the inlet air temperature and relative humidity changing curves. The set point was 15 °C/60% and 35 °C/61% for (a) and (b), with air flow rates of 0.03 m·s^−1^. From the figure, we could get that the inlet air temperature and relative humidity could be controlled around the set points. The average control error of inlet air temperature was within ±1 °C, and ±10% for relative humidity control. The adjusting time was about 15 min before the controlled variable got into the control range of set point in both Figure 8a,b. Similar dynamic and static control characteristics were observed in Figure 9 and Figure 10. The results showed that this platform could perform well during the inlet air temperature and relative humidity control. This platform could be used in various aeration conditions, such as in-bin cooling, drying or even rewetting, with good control performance.

### 4.2. Real-Time Data Acquisiton of the Stored Grain Bulk Test Results and Discussion

**Figure 11:**
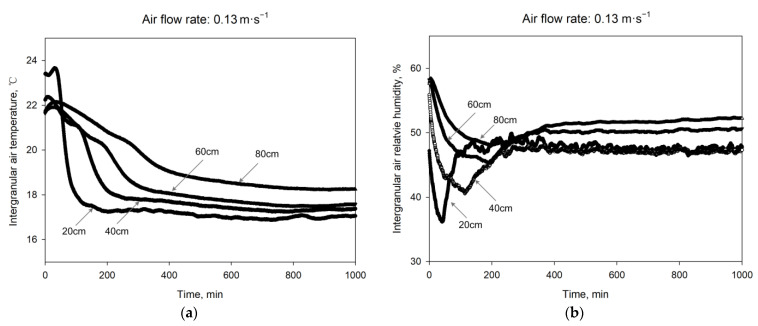
Real-time data acqusition test results of stored grain aeration: (**a**) Intergranular air temperature changing curves at different heights; (**b**) iIntergranular air relative humidity changing curves at different heights.

Intergranular air temperature and relative humidity of the stored grain at different heights (20 cm, 40 cm, 60 cm, 80 cm) were acquired during aeration which last 1000 min. The results were shown in Figure 11. Intergranular air temperature and relative humidity parameters acquired by sensors of different heights obtained near to the set point of inlet air temperature gradually, and those near to the inlet changed first and more sensitive to the inlet air change. Different time delays of changing trends were observed at different heights, which was in accorded with the phenomenon in real aerated bins. During the processing of aeration, the distance between the sensor and the air inlet increased, and the larger temperature deviation was observed, which was resulted from the grain thermal inertia. In real aeration process, hysteresis existed between the grain desorption and adsorption state, so the relative humidity did not follow the equilibrium moisture content Equation (2) all the time. The results showed that the four sensors fixed at different height inside the bin for intergranular air temperature and relative humidity monitoring worked well and managed to acquired data for aeration analysis.

## 5. Conclusions

Experiments provided effective means for the stored grain aeration mechanism and process control studies. This paper presented a new multi-functional experimental platform for stored grain aeration, and introduced a ON/OFF-PID based multivariable cooperative control method and the real-time data acquisition system in detail. Validation tests were carried out that showed the average control errors were acceptable. The aeration process could be monitored and real-time data of parameters of both the inlet air and intergranular air inside the grain bulk was acquired. All the data could be displayed on a touch panel and stored for analysis. Laboratory-scale aeration experiments of different objectives such as cooling, drying or rewetting of the grain for mechanism or control study could be executed on this platform. Moreover, this platform was compact, low-cost, easy to operate and more automatic compared with the present work. To improve the accuracy of inlet air temperature and relative humidity, more advanced control method will be needed. Moreover, more sensors could be placed inside the bin to better understand the mechanism of stored grain aeration.

## Data Availability

Not applicable.
